# Effectiveness of Yoga for Menopausal Symptoms: A Systematic Review and Meta-Analysis of Randomized Controlled Trials

**DOI:** 10.1155/2012/863905

**Published:** 2012-11-07

**Authors:** Holger Cramer, Romy Lauche, Jost Langhorst, Gustav Dobos

**Affiliations:** Chair of Complementary and Integrative Medicine, University of Duisburg-Essen, Essen, Germany

## Abstract

*Objectives*. To systematically review and meta-analyze the effectiveness of yoga for menopausal symptoms. *Methods*. Medline, Scopus, the Cochrane Library, and PsycINFO were screened through April 2012. Randomized controlled trials (RCTs) were included if they assessed the effect of yoga on major menopausal symptoms, namely, (1) psychological symptoms, (2) somatic symptoms, (3) vasomotor symptoms, and/or (4) urogenital symptoms. For each outcome, standardized mean differences (SMDs) and 95% confidence intervals (CIs) were calculated. Two authors independently assessed risk of bias using the risk of bias tool recommended by the Cochrane Back Review Group. *Results*. Five RCTs with 582 participants were included in the qualitative review, and 4 RCTs with 545 participants were included in the meta-analysis. There was moderate evidence for short-term effects on psychological symptoms (SMD = −0.37; 95% CI −0.67 to −0.07; *P* = 0.02). No evidence was found for total menopausal symptoms, somatic symptoms, vasomotor symptoms, or urogenital symptoms. Yoga was not associated with serious adverse events. *Conclusion*. This systematic review found moderate evidence for short-term effectiveness of yoga for psychological symptoms in menopausal women. While more rigorous research is needed to underpin these results, yoga can be preliminarily recommended as an additional intervention for women who suffer from psychological complaints associated with menopause.

## 1. Introduction

Menopause is defined as the permanent cessation of ovarian function and is thereby the end of a woman's reproductive phase [1]. Menopause begins around the age of 50 years [2] and is characterized by at least 12 months of amenorrhea [3]. While it is an inevitable part of every woman's life, about 3 out of every 4 women experience complaints during menopause [4, 5]. The most common menopausal symptoms include hot flashes, night sweats, fatigue, pain, decreased libido, and mood changes [6–8]. These symptoms often persist for several years postmenopause [9]. While hormone replacement therapy can effectively reduce menopausal symptoms [10, 11], its safety has long been controversially discussed [10–12]. While the most recent research has shown relatively low risk of severe adverse events for hormone replacement within the first 10 years of menopausal onset [10, 11, 13], the long-standing uncertainty about its safety has nevertheless led to significant decreases in hormone prescriptions to menopausal women [14]. Nonhormonal pharmacologic therapies have been shown to be less effective than hormonal therapy and to be associated with their own adverse events that restrict their use for many women [15]. Therefore, many menopausal women use complementary therapies to cope with their symptoms [16–21], and yoga is among the most commonly used complementary therapies for menopausal symptoms [18–21]. 

An estimated 15 million American adults report having practiced yoga at least once in their lifetime, almost half of those using yoga explicitly for coping with symptoms or promoting health [22]. Deriving from ancient Indian philosophy, yoga comprises physical postures as well as advice for ethical lifestyle and spiritual practice with the ultimate goal of uniting mind, body, and spirit [23, 24]. In North America and Europe, yoga is most often associated with physical postures (asana), breathing techniques (pranayama), and meditation (dyana) [24]. A variety of yoga schools have evolved from the traditional Indian system of yoga in Western societies, which are giving different weight to physical and spiritual practice [24]. Yoga interventions have been shown to decrease anxiety [25], distress [26], blood pressure [26], pain [27, 28], and fatigue [29].

A systematic review that included studies until mid of 2008 concluded that the evidence was insufficient to recommend yoga as an intervention for menopausal symptoms [30]. In the meantime, further large studies on yoga for menopausal symptoms have been published. Therefore, the aim of this paper was to systematically assess and meta-analyze the effectiveness of yoga in women with menopausal symptoms.

## 2. Methods

The review was planned and conducted in accordance with PRISMA guidelines for systematic reviews and meta-analyses [31] and the recommendations of the Cochrane Collaboration [32, 33].

### 2.1. Literature Search

The literature search comprised the following electronic databases from their inception through April 2012: Medline (via Pubmed), Scopus, the Cochrane Library, and PsycINFO. The literature search was constructed around search terms for “yoga” and search terms for “menopause” and adapted for each database as necessary. The complete search strategy for Pubmed was as follows: (“Yoga”[Mesh] OR yog *[Title/Abstract]) AND (“Climacteric”[Mesh] OR “Menopause”[Mesh] OR “Postmenopause”[Mesh] OR “Perimenopause”[Mesh] OR “Hot Flashes”[Mesh] OR menopaus *[Title/Abstract] OR peri-menopaus *[Title/Abstract] OR perimenopaus *[Title/Abstract] OR post-menopaus *[Title/Abstract] OR post-menopaus *[Title/Abstract] OR climact *[Title/Abstract] OR hot-flash *[Title/Abstract] OR hot flash *[Title/Abstract] OR hot-flush *[Title/Abstract] OR hot flush *[Title/Abstract] OR night sweat *[Title/Abstract]). Additionally, reference lists of identified original and review papers and the table of contents of the *International Journal of Yoga Therapy* and *Yoga Therapy Today* were searched manually.

Abstracts identified during literature search were screened, and the full articles of potentially eligible studies were read in full by 2 authors to determine whether they met the inclusion criteria.

### 2.2. Inclusion Criteria

To be eligible, studies had to meet the following conditions.

1. *Types of studies*. Randomized controlled trials (RCTs) were eligible. Studies were eligible only if they were published as full paper in English, German, French, or Norwegian language.
2. *Types of participants*. Studies of adult women who were experiencing menopausal or postmenopausal symptoms were eligible.
3. *Types of interventions*. Studies that compared yoga interventions with no treatment or any active treatment were eligible. Studies were excluded if yoga was not the main intervention but a part of a multimodal intervention, such as mindfulness-based stress reduction. Since in North America and Europe, physical exercise is perceived as a main component of yoga [24], studies examining meditation or yogic lifestyle without physical component were also excluded. No further restrictions were made regarding yoga tradition, length, frequency or duration of the program. Cointerventions were allowed.
4. *Types of outcome measures*. Studies were eligible if they assessed major menopausal symptoms, namely, (1) psychological symptoms (e.g., depression, anxiety, sleep disorders), (2) somatic symptoms (e.g., pain, fatigue), (3) vasomotor symptoms (e.g., hot flashes, night sweats), and/or (4) urogenital symptoms (e.g., sexual dysfunctions, bladder problems).

### 2.3. Data Extraction

Two reviewers independently extracted data on characteristics of participants (e.g., sample size, inclusion criteria, age), characteristics of the intervention and control (e.g., yoga tradition, program length, frequency and duration), and outcome measures and results. If necessary, discrepancies were rechecked with a third reviewer and consensus achieved by discussion.

#### 2.3.1. Risk of Bias in Individual Studies

Risk of bias was assessed by 2 reviewers independently using the 12 criteria (rating: yes, no, unclear) recommended by the Cochrane Back Review Group [33]. These criteria assess risk of bias on the following domains: selection bias, performance bias, attrition bias, reporting bias, and detection bias. If necessary, discrepancies were rechecked with a third reviewer and consensus achieved by discussion. Studies that met at least 6 of the 12 criteria and had no serious flaw were rated as having low risk of bias. Studies that met fewer than 6 criteria or had a serious flaw were rated as having high risk of bias.

### 2.4. Data Analysis

Studies were analyzed separately for short- and long-term followups. For the purpose of this review, short-term followup was defined as the outcome measures taken closest to 12 weeks after randomization and long-term followup as measures taken closest to 12 months after randomization [33].

### 2.5. Assessment of Overall Effect Size

Meta-analyses were calculated using Review Manager 5 software (Version 5.1, The Nordic Cochrane Centre, Copenhagen) if at least 2 studies on a specific outcome were available. If studies had two or more control groups, the control groups for assessment of overall effect were selected in the following order of preference: no treatment, attention control, and active comparator. A random effects model was used because it involves the assumption of statistical heterogeneity between studies [32]. Standardized mean differences (SMD) with 95% confidence intervals (CI) were calculated as the difference in means between groups divided by the pooled standard deviation. Where no standard deviations were available, they were calculated from standard errors, confidence intervals or t values [32]; or attempts were made to obtain the missing data from the trial authors by email. The magnitude of the effect size was calculated using Cohen's categories with (1) SMD = 0.2–0.5: small; (2) SMD = 0.5–0.8: moderate; (3) SMD > 0.8: large effect sizes [34]. 

Levels of evidence were determined according to previously published recommendations with (1) strong evidence: consistent findings among multiple RCTs with low risk of bias; (2) moderate evidence: consistent findings among multiple high-risk RCTs and/or one low-risk RCT; (3) limited evidence: one RCT with high risk of bias; (4) conflicting evidence: inconsistent findings among multiple RCTs; (5) No evidence: no RCTs [35].

### 2.6. Assessment of Heterogeneity

Statistical heterogeneity between studies was quantified using the *I* ^2^ statistics, a measure of how much variance between studies can be attributed to differences between studies rather than chance. The following categories were used to calculate the magnitude of heterogeneity: *I* ^2^ = 0–30%: no heterogeneity; *I* ^2^ = 30–49%: moderate heterogeneity, *I* ^2^ = 50–74%: substantial heterogeneity; *I* ^2^ = 75–100%: considerable heterogeneity [32]. The Chi^2^ test was used to assess whether differences in results are compatible with chance alone. A *P* value ≤ 0.10 was regarded to indicate significant heterogeneity [32].

### 2.7. Subgroup and Sensitivity Analyses

Subgroup analyses were planned for type of control intervention (no treatment; attention control; active comparator). To test the robustness of significant results, sensitivity analyses were conducted for studies with high versus low risk of bias. 

If statistical heterogeneity was present in the respective meta-analysis, subgroup and sensitivity analyses were also used to explore possible reasons for heterogeneity.

### 2.8. Risk of Bias across Studies

If at least 10 studies were included in a meta-analysis, funnel plots were generated using Review Manager 5 software. Publication bias was assessed by visual analysis with roughly symmetrical funnel plots regarded to indicate low risk and asymmetrical funnel plots regarded to indicate high risk of publication bias [36].

## 3. Results

### 3.1. Literature Search

**Figure 1:**
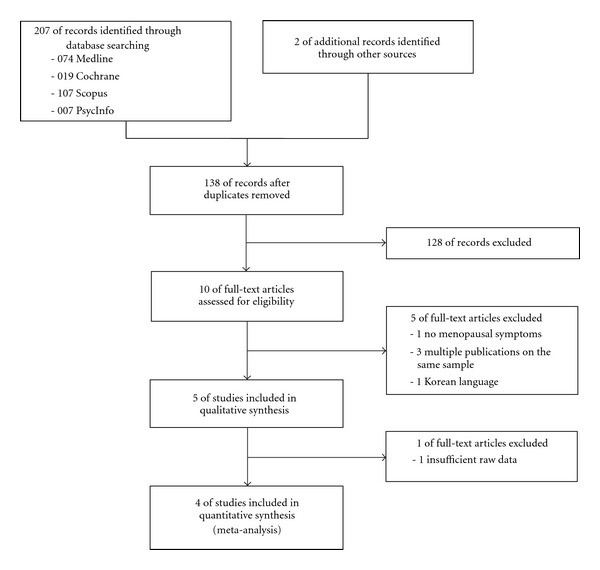
Flowchart of the results of the literature search.

The literature search revealed a total of 207 records. One additional record each was found in reference lists of identified review papers and the table of contents of the *International Journal of Yoga Therapy*, respectively. Seventy-one duplicates were excluded. Further 128 records were excluded since they did not meet all inclusion criteria. Ten full-text articles were assessed [37–46] and 5 articles were excluded; 1 article did not assess menopausal symptoms [37], 1 RCT was published in Korean language [38], and 3 articles reported additional outcome measures for already included studies on the same participant population [39–41]. Therefore, 5 RCTs with a total of 582 participants [42–46] were included in qualitative analysis. One RCT did not report standard deviations, nor standard errors, confidence intervals or *t*-values [43]. Since the missing data could not be obtained from the authors of the respective study by email, this study was excluded from quantitative analysis. Finally, 4 RCTs with overall 545 participants were included in the meta-analysis (Figure 1).

### 3.2. Study Characteristics

Characteristics of the sample, interventions, outcome assessment, and results are shown in Table 1. In the following, study characteristics will be presented for all trials included in qualitative synthesis.

#### 3.2.1. Setting and Participant Characteristics

Two RCTs originated from the USA [43, 45], 1 from Brazil [42] and 2 from India [44, 46]. Studies were conducted at university medical centers [37, 38, 40] or yoga institutes [44, 46]. Patients were recruited from university medical center oncology units [43] and gynecological outpatient clinics [42, 44] or by using advertisements [42, 44–46]. 

Between 31.9% and 47.2% of women in each RCT were postmenopausal (median: 46.4%). One study included only women who experienced menopausal symptoms after breast cancer treatment [43]. Participants' mean age ranged from 45.6 years to 54.9 years with a median of 49.0 years. Between 0% and 82.6% of included participants were Caucasians with a median of 81.1%.

#### 3.2.2. Intervention Characteristics

Yoga traditions were heterogeneous between studies: 1 RCT each used Iyengar yoga [45]; an integrated approach to yoga therapy [44]; yoga of awareness [43]; a combination of Yogasana and Tibetan yoga [42]; traditional Indian yoga [46], respectively. All yoga programs included yoga postures and meditation/relaxation; 4 programs also encompassed breathing techniques [42–44, 46]. Program length and intensity varied, ranging from weekly sessions over 8 weeks [43] to daily sessions over 90 days [46]. Generally, frequency of interventions was much higher in the Indian studies [44, 46] compared to the studies conducted in North or South America [42, 43, 45]. Yoga was taught by at least 1 certified and experienced yoga teacher in 2 trials [43, 45], while 3 studies did not state qualification of yoga teachers [42, 44, 46]. 

Two RCTs compared yoga to no treatment [43, 46]; 1 RCT compared yoga to exercise [44]; 2 3-arm RCTs compared yoga to no treatment and exercise [42, 45]. Program length, frequency, and duration of the exercise control arms were exactly matched with the yoga interventions in 2 studies [42, 44], while the yoga and exercise intervention in the third RCT were matched for total contact time [45].

#### 3.2.3. Outcome Measures

Total menopausal symptoms were assessed in 3 studies using the Kupperman Menopausal Index [42], the Greene Climacteric Scale [45], or the Menopause Rating Scale [46]. Psychological symptoms were assessed in all 5 RCTs using either menopause-specific scales [43, 44, 46], generic questionnaires [42], or both [45]. Using menopause-specific scales, somatic symptoms were assessed by 4 RCTs [43–46]; vasomotor symptoms by 3 RCTs [43–45]; urogenital symptoms by 2 RCTs [45, 46]. Only 1 RCT reported safety data [42].

#### 3.2.4. Risk of Bias in Individual Studies

Two RCTs had low risk of bias [43, 46] and 3 RCTs had high risk of bias [42, 44, 45] (Table 2). Risk of selection bias mainly was low; all but 1 RCT [42] reported adequate randomization, while only 2 RCTs reported adequate allocation concealment [43, 46]. No study reported blinding of participants or providers, while 2 studies reported adequate blinding of outcome assessors [43, 45]. Only 1 study included an adequate intention-to-treat analysis [43]. Risk of selective outcome reporting was high in 2 studies that reported different outcomes from the same RCT in multiple publications without disclosing the entire study protocol [44, 45].

### 3.3. Analyses of Overall Effects

**Figure 2:**
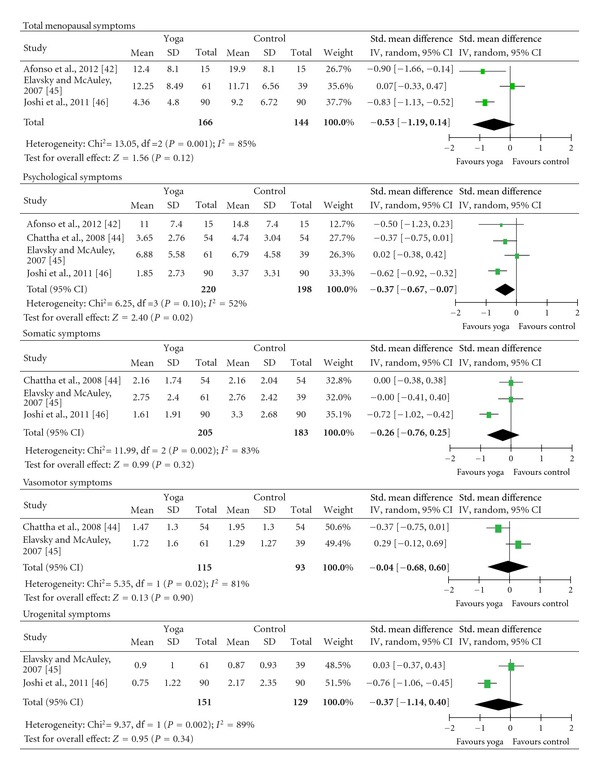
Forest plots of overall short-term effects of yoga on menopausal symptoms.

Meta-analyses did not reveal evidence for short-term effects on total menopausal symptoms (SMD = −0.53; 95% CI −1.19 to 0.14; *P* = 0.12; heterogeneity: *I* ^2^ = 85%; Chi² = 13.05; *P* < 0.01). Moderate evidence was found for short-term effects on psychological symptoms (SMD = −0.37; 95% CI −0.67 to −0.07; *P* = 0.02; heterogeneity: *I* ^2^ = 52%; Chi² = 6.25; *P* = 0.10). Based on Cohen's categories, the effects on psychological symptoms were of small size. There was no evidence for short-term effects on somatic symptoms (SMD = −0.26; 95% CI −0.76 to 0.25; *P* = 0.32; heterogeneity: *I*² = 83%; Chi² = 11.99; *P* < 0.01), vasomotor symptoms (SMD = −0.04; 95% CI −0.68 to 0.60; *P* = 0.90; heterogeneity: *I*² = 81%; Chi² = 5.35; *P* = 0.02), or urogenital symptoms (SMD = −0.37; 95% CI −1.14 to 0.40; *P* = 0.34; heterogeneity: *I*² = 89%; Chi² = 9.37; *P* < 0.01) (Figure 2).

Only 1 RCT included a longer-term followup for yoga compared to no treatment. At 20-week followup, this study reported significant group differences for psychological, somatic, and vasomotor symptoms [43] (Table 1).

Only 1 RCT included safety data and reported that yoga was not associated with any adverse events [42].

#### 3.3.1. Subgroup and Sensitivity Analyses

When comparing yoga to no treatment, there was no evidence for short-term effects on total menopausal symptoms, psychological symptoms, somatic symptoms, or urogenital symptoms (Table 3). When comparing yoga to exercise, there was no evidence for short-term effects on total menopausal symptoms, psychological symptoms, somatic symptoms, vasomotor symptoms, or urogenital symptoms (Table 3).

Since only 1 RCT with low risk of bias was included in the meta-analyses, no formal sensitivity analysis of the effects on psychological symptoms was possible. However, the RCT with low risk of bias [46] had higher effect size and narrower confidence intervals than the RCTs with high risk of bias [42, 44, 45] (Figure 2).

#### 3.3.2. Risk of Bias across Studies

Since less than 10 studies were included in each meta-analysis, funnel plots were not analyzed.

### 3.4. Discussion

This systematic review found moderate evidence for short-term improvements of psychological symptoms in menopausal women after yoga interventions. However, no evidence was found for improvements regarding somatic, vasomotor, urogenital, or total menopausal symptoms. Further, no group difference was found when comparing yoga to exercise. The available safety data suggest that yoga is not associated with serious adverse events. However, future RCTs should ensure more rigorous reporting of adverse events and reasons for dropouts.

The conclusions of the present review are not in line with a recent qualitative systematic review on mind-body interventions, which concluded that there was moderate evidence that yoga might relief common menopausal symptoms including vasomotor and psychological symptoms [47]. On the other hand, the finding of a small significant reduction of psychological symptoms in the present review is also not in line with another systematic review that concluded that yoga is ineffective in relieving any menopausal symptoms including psychological symptoms [30]. Differences in inclusion criteria such as the inclusion of nonrandomized studies in both aforementioned reviews as well as new evidence that is now available [42, 43, 46] might at least partly explain the differences in results. Therefore, the latter review included much less RCTs and participants than the present one; for example, 2 RCTs and 232 participants in the meta-analysis on psychological symptoms [30], compared to 4 RCTs and 418 participants in the present review. The results of the present review are, however, in line with those of a Cochrane review that found no differences between yoga and exercise in vasomotor symptom relief [48].

#### 3.4.1. External and Internal Validity

Patients in the included studies were recruited from inpatient clinics, outpatient clinics, and yoga centers and by advertisements in North America, South America, and Asia. Patients' age ranged from their mid-forties to their mid-fifties and members of different ethnic groups were included. Most studies included peri- or postmenopausal women that were healthy besides their menopausal symptoms; however, 1 study specifically included only breast cancer survivors [43]. The results of this review are therefore applicable to the vast majority of women with menopausal symptoms in clinical practice. External validity is however limited by the high frequency of yoga sessions especially in the 2 Indian studies [44, 46]. Yoga programs that require daily meetings over several weeks might be hard to establish in Western societies.

Three out of 5 studies had high risk of bias [42, 44, 45]. One of the 2 studies with low risk of bias [43, 46] could not be included in the meta-analysis [43]. Adequate allocation concealment was reported in only 2 studies, [43, 46], and only 2 studies reported adequate blinding of outcome assessors [43, 45]. Blinding patients or care providers in yoga studies might not be possible at all [27]. No study that was included in the meta-analysis applied adequate intention-to-treat analysis. While no formal sensitivity analysis was possible, the only RCT with low risk of bias that could be included in the meta-analysis for psychological symptoms [46] had higher effect size and narrower confidence interval than the RCTs with high risk of bias. While the effects of yoga on psychological symptoms therefore seem to be robust against bias, definite judgments on internal validity of the results cannot be drawn.

#### 3.4.2. Strengths and Weaknesses

This review is the first meta-analysis available on menopausal symptoms that included only randomized controlled trials. Moreover, in contrast to the only other available meta-analysis on yoga for menopausal symptoms [30], subgroup analyses for different types of control interventions were possible for almost all prespecified outcome measures, and the levels of evidence were determined according to established recommendations [35].

The primary limitation of this paper is the small total number of eligible RCTs. Moreover, since only 1 study included a longer-term followup, no formal meta-analysis on long-term effects of yoga for menopausal symptoms was possible. The overall high risk of bias further restricts the interpretation of the results. Substantial statistical heterogeneity was present in the significant meta-analysis on psychological symptoms and subgroup analysis could not provide reasons for heterogeneity. Further limitations include the restriction of eligible publication languages, and the use of compound scores for psychological symptoms in most of the included studies. Therefore, the specific variables that were improved by the yoga interventions, for example, depression, anxiety, or sleep, could not be evaluated.

#### 3.4.3. Implications for Further Research

Given the popularity of yoga among patients with menopausal symptoms, further studies are warranted. These studies should ensure rigorous methodology and reporting, mainly adequate randomization, allocation concealment, intention-to-treat analysis, and blinding of at least outcome assessors [49]. Comparisons of yoga to adequately matched active control interventions are equally needed as comparisons of different yoga styles. Dismantling studies that separately evaluate the effects of different components of yoga such as physical postures, breathing techniques, or meditation would further improve knowledge of the underlying mechanisms of yoga in menopausal symptom relief.

## 4. Conclusion

This systematic review found moderate evidence for short-term effectiveness of yoga for psychological symptom relief in menopausal women. Since many menopausal women request complementary therapies either instead of hormone replacement therapy or in addition to it, yoga can be preliminarily recommended as an adjunct intervention for women who suffer from psychological complaints associated with menopause. However, more rigorous research is needed to underpin these results.

## Figures and Tables

**Table 1:** Characteristics of the included studies.

Author, year	Sample size, no. of groups	Mean age ± standard deviation	Inclusion criteria	Treatment group: Intervention Program length, frequency, duration	Control group: Intervention Program length, duration, frequency	Outcome assessment (a) Short-term followup (b) Long-term followup	Outcome measures (1) Total menopausal symptoms (2) Psychological symptoms (3) Somatic symptoms (4) Vasomotor symptoms (5) Urogenital symptoms (6) Safety	Results^a^ (a) Short-term followup (b) Long-term followup (1) Total menopausal symptoms (2) Psychological symptoms (3) Somatic symptoms (4) Vasomotor symptoms (5) Urogenital symptoms (6) Safety
Afonso et al., 2011 [42]	*N* = 61, 3 groups	NR	Postmenopausal women (50–65 years) Diagnosed insomnia not due to dyspnea	Yogasana and Tibetan yoga: yoga postures, breathing techniques, relaxation 4 months, twice weekly, 60 minutes	(1) Passive stretching 4 months, twice weekly, 60 minutes (2) Wait-list, no treatment 4 months	(a) month 4 (b) NA	(1) Total menopausal symptoms (KMI) (2) Anxiety (BAI), depression (BDI), insomnia (ISI) (3) NA (4) NA (5) NA (6) Safety	(a) (1) Yoga < wait-list (2) BAI: NS, BDI: NS, ISI: Yoga < wait-list (3) NA (4) NA (5) NA (b) NA (6) No adverse events

Carson et al., 2009 [43]	*N* = 37, 2 groups	Yoga: 53.9 ± 9.0 years Control: 54.9 ± 6.2 years	Breast cancer survivors ≥ 1 hot flash/day on ≥ 4 days/week No hormone replacement therapy within last 3 months No regular yoga	Yoga of awareness: yoga postures, breathing techniques, meditation, study of pertinent topics, group discussion 8 weeks, once weekly, 120 minutes	Wait-list, no treatment 8 weeks	(a) Week 6 (b) Week 20	Daily diary (numerical rating scale): (1) NA (2) Negative mood, sleep disturbances, bother (3) Joint pain, fatigue (4) Hot flashes (frequency, severity, total score), night sweats (5) NA (6) NA	(a) (1) NA (2) Negative mood: NS, sleep disturbances: Yoga < wait-list, bother: Yoga < wait-list (3) Joint pain: Yoga < wait-list, fatigue: Yoga < wait-list (4) Hot flashes frequency: Yoga < wait-list, severity: Yoga < wait-list, total score: Yoga < wait-list, night sweats: NS (5) NA (b) (1) NA (2) Negative mood: Yoga < wait-list, sleep disturbances: NA, bother: Yoga < wait-list (3) Joint pain: Yoga < wait-list, fatigue: Yoga < wait-list (4) Hot flashes frequency: Yoga < wait-list, severity: Yoga < wait-list, total score: Yoga < wait-list, night sweats: NS (5) NA (6) NA

Chattha et al., 2008 [44]	*N* = 120, 2 groups	Yoga: 49.0 ± 3.6 years Control: 48.0 ± 4.0 years	Women (45–55 years) with menopausal symptoms FSH level ≥ 15 mlU/ml No hormone replacement therapy	Integrated approach to yoga therapy: yoga postures, breathing techniques, meditation, lectures on lifestyle 8 weeks, 5 times weekly, 60 minutes	Exercise: walking, stretching, rest, lectures on lifestyle 8 weeks, 5 times weekly, 60 minutes	(a) Week 8 (b) NA	(1) NA (2) Psychological symptoms (GCS) (3) Somatic symptoms (GCS) (4) Vasomotor symptoms (GCS) (5) NA (6) NA	(a) (1) NS (2) NS (3) NS (4) Yoga < exercise (5) NS (b) NA (6) NA

Elavsky and McAuley, 2007 [45]	*N* = 164, 3 groups	Yoga: 50.0 ± 3.7 Walking: 50.5 ± 3.4 Wait-list: 48.6 ± 3.5	Women (45–55 years) with menopausal symptoms No hormone replacement therapy	Iyengar yoga: yoga postures, meditation 4 months, twice weekly, 90 minutes	(1) Walking 4 months, 3 times weekly, 60 minutes (2) wait-list, no treatment 4 months	(a) Month 4 (b) NA	(1) Total menopausal symptoms (GCS) (2) Psychological symptoms (GCS), affect (Affectometer 2), Depression (BDI) (3) Somatic symptoms (GCS) (4) Vasomotor symptoms (GCS) (5) Urogenital symptoms (GCS) (6) NA	(a) (1) NS (2) GCS: NS, Positive affect: Wait-list < yoga, BDI: NS (3) NS (4) NS (5) NS (b) NA (6) NA

Joshi et al., 2011 [46]	*N* = 200, 2 groups	Yoga: 45.6 ± 3.9 years Control: 46.3 ± 3.5 years	Women (40–55 years) with irregular cycle or postmenopausal No hormone replacement therapy No yoga practice	Yoga: yoga postures, breathing techniques, meditation 90 days, daily, 60 minutes	Wait-list, no treatment 90 days	(a) Day 90 (b) NA	(1) Total menopausal symptoms (MRS) (2) Psychological symptoms (MRS) (3) Somatovegetative symptoms (MRS) (4) NA (5) Urogenital symptoms (MRS) (6) NA	(a) (1) Yoga < wait-list (2) Yoga < wait-list (3) Yoga < wait-list (4) NA (5) Yoga < wait-list (b) NA (6) NA

Abbreviations: BAI: beck anxiety inventory; BDI: beck depression inventory; GCS: Greene climacteric scale; ISI: insomnia severity index; KMI: Kupperman menopausal index; MRS: menopause rating scale; NA: not applicable; NS: not significant.

^a^< indicates significantly lower scores.

**Table 2:** Risk of bias assessment of the included studies using the Cochrane Back Review Group risk of bias tool.

Author, year	Bias												
	Selection bias:			Performance bias:				Attrition bias:		Reporting bias:	Detection bias:		Total: (max. 12)^a^
	Adequate random sequence generation	Adequate allocation concealment	Similar baseline characteristics	Adequate participant blinding	Adequate provider blinding	Similar or no co-interventions	Acceptable compliance	Acceptable and described drop-out rate	Inclusion of an intention-to-treat analysis	No selective outcome reporting	Adequate outcome assessor blinding	Similar timing of outcome assessment	
Afonso et al., 2011 [42]	Unclear	Unclear	No	Unclear	Unclear	Unclear	Unclear	No	No	Yes	Unclear	Yes	2
Carson et al., 2009 [43]	Yes	Yes	Yes	Unclear	Unclear	Yes	Yes	Yes	Yes	Yes	Yes	Yes	10
Chattha et al., 2008 [44]	Yes	Unclear	Yes	No	No	Unclear	Yes	Yes	No	No	Unclear	Yes	5
Elavsky and McAuley, 2007 [45]	Yes	Unclear	No	Unclear	Unclear	Unclear	Yes	Yes	No	No	Yes	Yes	5
Joshi et al., 2011 [46]	Yes	Yes	Yes	Unclear	Unclear	Unclear	Yes	Yes	No	Yes	Unclear	Yes	7

^a^Higher scores indicate lower risk of bias.

**Table 3:** Subgroup analyses: effect sizes of yoga versus controls.

Outcome	No. of studies	No. of patients	No. of patients	Standardized mean difference	*P*	Heterogeneity
		(yoga)	(control)	[95% confidence interval]	(overall effect)	*I* ^2^; Chi^2^; *P*
Yoga versus no treatment^a^						
Total symptoms	3	166	144	−0.53 [−1.19, 0.14]	0.12	85%; 13.05; <0.01
Psychological symptoms	3	166	144	−0.36 [−0.81, 0.09]	0.12	68%; 6.23; 0.04
Somatic symptoms	2	151	129	−0.38 [−1.08, 0.33]	0.29	87%, 7.86; <0.01
Vasomotor symptoms	—	—	—	—	—	—
Urogenital symptoms	2	151	129	−0.37 [−1.14, 0.40]	0.34	89%; 9.37; <0.01
Yoga versus exercise^a^						
Total symptoms	2	76	77	0.10 [−0.37, 0.58]	0.67	38%; 1.61; 0.20
Psychological symptoms	3	130	131	0.10 [−0.43, 0.62]	0.72	75%; 7.93; 0.02
Somatic symptoms	2	115	117	0.06 [−0.20, 0.32]	0.66	0%; 0.17; 0.68
Vasomotor symptoms	2	115	117	−0.13 [−0.58, 0.33]	0.58	67%, 3.07; 0.08
Urogenital symptoms	2	151	129	−0.37 [−1.14, 0.40]	0.34	89%; 9.37; <0.01

^a^Reference [42, 45] with one control arm each.
